# Right hemisphere occipital rTMS impairs working memory in visualizers but not in verbalizers

**DOI:** 10.1038/s41598-019-42733-6

**Published:** 2019-04-19

**Authors:** Sven Hilbert, Michaela McAssey, Markus Bühner, Patrick Schwaferts, Monika Gruber, Stephan Goerigk, Paul Christopher John Taylor

**Affiliations:** 1 Faculty of Psychology, Educational Science, and Sport Science, University of Regensburg, Universitätsstraße 31, 93053 Regensburg, Germany; 2 Department of Neurology, University Hospital, LMU Munich, Munich, Germany; 3 Graduate School of Systemic Neuroscience, Research Training Group 2175, Ludwig-Maximilians-University, Leopoldstraße 13, 80802 München, Germany; 4 Department of Psychology, Psychological Methods and Assessment, LMU Munich, Leopoldstraße 13, 80802 München, Germany; 5 Institute of Statistics, Methodological Foundations of Statistics and its Applications, Ludwigstraße 33, 80539 München, Germany; 6 Department of Psychiatry and Psychotherapy, Ludwig-Maximilians-University, Nußbaumstraße 7, 80336 Munich, Germany; 7 Hochschule Fresenius, University of Applied Sciences, Infanteriestraße 11A, 80797 Munich, Germany; 8 German Center for Vertigo and Balance Disorders, University Hospital, LMU Munich, Munich, Germany

**Keywords:** Sensory processing, Human behaviour

## Abstract

Distinguishing between verbal and visual working memory processes is complicated by the fact that the strategy used is hard to control or even assess. Many stimuli used in working memory tasks can be processed via verbal or visual coding, such as the digits in the digit span backwards task (DSB). The present study used repetitive transcranial magnetic stimulation (rTMS) to examine the use of visual processing strategies in the DSB. A total of 47 German university students took part in the study, 23 spontaneously using a verbal processing strategy and 24 using a visual strategy. After rTMS to the right occipital cortex, visualizers showed a significantly stronger mean performance decrease compared to verbalizers. The results indicate that the visual cortex is more critical for visualizers compared to verbalizers in the DSB task. Furthermore, the favored processing modality seems to be determined by the preference for a cognitive strategy rather than the presentation modality, and people are aware of the applied strategy. These findings provide insight into inter-individual differences in working memory processing and yield important implications for laboratory studies as well as clinical practice: the stimulus does not necessarily determine the processing and the participant can be aware of that.

## Introduction

Working memory (WM) processes describe a cognitive system responsible for temporary storage and manipulation of information. Temporarily holding information available is crucial to coordinate processing when multiple goals are active in complex cognitive tasks such as language transformation, reasoning, or reading^1^. Various conceptional approaches have been proposed to model the processing involved in this kind of task^2^. Recently, a class of state-based models, that are well supported by neuroimaging and neurophysiological data, have gained in prominence. In these models, the allocation of attention to internal representations plays a crucial role for the retention of information in WM. Notable models include those proposed by Cowan^3^, Oberauer^4,5^, or McElree^6,7^. For a comprehensive review on state-based models, see D’Esposito and Postle^8^. Preceding most state-based conceptions of working memory, Baddeley & Hitch^9^ proposed one of the last century’s most significant models. It includes a master system, the central executive, and two slave systems, termed “visuospatial sketchpad” and “phonological loop”, which are thought to be involved in verbal and visual working memory tasks, respectively.

Despite the differences between models regarding the exact organization of working memory^10^, all widely agree that it is partly defined by its limited capacity which varies between individuals^2,11^. Therefore, several paradigms have been developed to test for inter-individual differences. The “digit span backwards” task (DSB) is used as part of various psychological tests, like the “Wechsler Adult Intelligence Scales”^12^, and remains, especially in clinical psychology, a common method for working memory assessment^8^. In order to relate the DSB (or any operationalization at all) to the concept of working memory, it is crucial to consider which cognitive processes can be used to form correct responses. Since a correct response in the DSB is defined by correctly repeating a successively presented digit sequence in reversed order, processing here involves the transformation of information that is needed to memorize, invert, and produce the digit series in reverse order. Two prominent strategies for doing so are (1) verbalizing all digits and repeating them silently and (2) mentally visualizing them and reading them backwards^13,14^. Verbal and visual processing strategies have been identified as the two most prominent approaches for cognitive tasks^15^ and people favoring one of the strategies can be divided into groups characterized by their typical processing^16^: verbalizers typically use articulatory techniques (i.e., inner rehearsal), while visualizers tend to adopt mental imagery. These strategies, naturally, go hand in hand with the visual and the verbal working memory subsystems outlined in Baddeley and Hitch’s model.

Importantly, even though everyday cognitive processing styles tend to be rigid and stable, task-specific strategies can be adopted rather flexibly to serve the task at hand via verbal or visual processing^17,18^. It has previously been assumed that the presentation mode during a task is critical for the adoption of a visual or verbal processing strategy^19–21^, while Hilbert *et al*.^14,17^ showed that participants use their preferred strategy independent from the acoustical or optical presentation mode. This differentiation is crucial, as the stimulus-independent use of processing strategies for working memory processing may not only perturb findings regarding visual and verbal working memory components but also has implications for clinical assessment.

Classically, memory consolidation and retrieval have been linked to neural areas outside the occipital visual sensory cortex. Various imaging studies have located the phonological loop’s neural correlates in the auditory cortex^22,23^ and the visuospatial sketchpad’s in occipito-parietal areas^21,24,25^. The central executive’s correlates have been reported in dorsolateral prefrontal cortex^2^, which is a cortical region related to complex cognitive processing^26^ and aids in the maintenance of information by directing attention to internal representations of sensory stimuli^27^. The possibility of localizing individual parts of the working memory system in the human cortex has led to various investigations studying the effects of stimulating separate components of the system: different forms of non-invasive brain stimulation (either transcranial magnetic stimulation (TMS) or transcranial current stimulation (tCS)) to the dorsolateral prefrontal cortex have been shown to enhance^28,29^ or deteriorate^30^ performance in working memory tasks. Similarly, the left temporo-parietal areas^31,32^ have been targeted via TMS to affect verbal working memory performance, while parietal stimulation can alter visual working memory performance^33^.

Of substantial recent interest is the growing evidence that early visual sensory areas are involved in the consolidation of visual working memory and TMS has contributed to this^34^. The precise coding and short-term storage of sensory representations has been postulated to be carried out in the respective sensory cortices^35–37^, marking working memory functioning as a cortical network comprising connected but dissociable units. In this vein, several groups have reported that occipital TMS affects performance during working memory tasks^38^. Occipital TMS pulses affect performance when applied immediately after stimulus onset (and the stimuli are presumably being stored into memory) but not during retention^39^, or only at the start and not end of a retention interval^40^. Online single pulse occipital TMS may only bear a disruptive effect during high memory load^41^. Single pulses of TMS applied after onset of an array of stimuli reduced performance mainly by increasing the probability of guessing, consistent with affecting primarily the quantity (capacity) and not quality (precision) of stored information^42^.

The present study uses these findings regarding differential stimulation of cortical regions involved in verbal and visual working memory processes to investigate the effect on processing strategies in the DSB. More specifically, a group of verbalizers and a group of visualizers both receive repetitive TMS (rTMS) to occipital cortex to create an offline virtual lesion before conducting the DSB. It is expected that, compared to a control condition, the deterioration in performance is stronger for visualizers than for verbalizers, as the latter are thought to rely less on visual cortex areas during working memory processing.

## Methods

### Sample

A total of 47 (31 female) university students (native German-speaking) took part in the investigation (median: 22; range 18–46 years). The participations received 25 Euros as gratification and a written confirmation of participation, exchangeable for course credit. To test an approximately even number of visualizers and verbalizers, all participants were screened for their strategy (using 8 digit series) and contacted later, depending on their strategy. Five subjects had to be excluded (4 due to phosphenes that impaired their vision during the experiment, 1 because the earplugs fell out and caused the experiment to stop). The final sample therefore consisted of 23 verbalizers and 24 visualizers.

### Materials

#### Assessment of digit span backwards performance

The stimulus material was presented in Arial font at a height of 3° of visual angle on a monitor. All stimuli were presented in black font on a white background. Each digit was presented for 1 s. The order of the digit series was randomized between the subjects. No digit appeared more than once in a single digit series. The keyboard of the computer was covered in order to prevent the subjects from using the number keys to assist memorization. In each testing session, the participant was presented with nine digit series: three with four digits, three with five digits, and three with six digits. Consequently, the maximum number of correctly remembered sequences was nine and the maximum number of correctly remembered digits was 45. After each digit series, the participants had time to repeat the digits vocally in reversed order and to prepare themselves for the upcoming series, adding up to a pause of about one minute between two digit series.

#### Assessment of cognitive strategies

After the tests had been conducted, the participants received a questionnaire about the cognitive strategies used to remember the digit series. First, the participants were asked to state how they solved the DSB task to help them to form a clear picture of how they solved the task. Afterwards, the participants had to decide in a two-alternative-forced-choice question whether they remembered the digits more visually or more verbally. The forced-choice question was used to categorize the participants into verbalizers and visualizers for the analysis.

### Procedure

The testing was performed in two separate sessions in a university laboratory under comparable conditions. The second session was conducted at least seven days after the first one to reduce retest effects. Half of the participants faced the control condition during the first session, the other half faced the experimental condition in the first session.

After the presentation of each digit sequence, the subjects were asked to vocally repeat the presented sequence in reversed order. For every digit, it was recorded if it had been remembered correctly or incorrectly.

#### Transcranial Magnetic Stimulation

In the experimental condition subthreshold offline TMS was applied to occipital cortex at 1 Hz for ten minutes using a MagPro R30 Stimulator (MagVenture, Denmark) with a MC B65-HO figure-of-8 coil: all methods were carried out in accordance with relevant guidelines^43^. The protocol was approved by the ethics commission of the German Psychological Association (DGPs), and all subjects gave informed consent. The TMS protocol used one of the two main forms of offline TMS, a 1 Hz protocol, which as reviewed by Parkin *et al*.^44^ is “widely used as an inhibitory intervention”^45^ and is classically associated with mimicking the effects of neuropsychological patients^46^. One Hz TMS was delivered for ten minutes as has been used successfully in previous studies of visual cognition^47,48^.

Determining TMS intensity used a three-step procedure. This procedure was necessary to account for interindividual variability, where some people see phosphenes from TMS pulses and some people do not, and that the safety guidelines^40^ specify motor threshold and not phosphene threshold. Firstly, for participants for whom it was possible to elicit phosphenes after occipital cortex stimulation, the occipital site was defined as the location over the right occipital lobe that resulted in the brightest and largest central phosphene. Phosphene thresholds were determined under conditions comparable to the main experiment, with eyes open, fixation on the center of the computer monitor, and the same lighting conditions. To determine phosphene threshold, stimulation intensity was decreased in steps of 2% until a phosphene was reported after five out of ten consecutive pulses. Offline TMS was then applied at 80% of this phosphene threshold to prevent phosphenes from being seen during the stimulation session and so make the participants’ experience during active and control stimulation tests comparable.

Secondly, if the derived phosphene threshold was more than 110% of active motor threshold then this would have exceeded the safety guidelines: therefore, stimulation intensity was reduced to 110% of the active motor threshold if that was lower than 80% phosphene threshold (this occurred in approximately equal numbers of visualizers and verbalizers, 5 and 6 respectively).

Thirdly, if it was not possible to elicit phosphenes in the participant, then a location 2 cm dorsal and 1 cm lateral to right of the inion was used with 90% of the right hemisphere (left hand) active motor threshold. The number of phosphene seers in the visualizer group was approximately equal to that in the verbalizer group (10 visualizers versus 13 verbalizers: this lack of a large difference between numbers of verbalizers and visualizers that see phosphenes, although potentially interesting for further work, was not a primary objective of this study).

Importantly, after our three-step intensity-determining algorithm, the visualizers and the verbalizers were stimulated at highly comparable intensities (visualizers: 40.4%; verbalizers 40.9%).

In the control condition TMS was applied with the same intensity within each participant but to the right sensorimotor cortex at a point lateral (to the right) of the vertex at the same distance from the midline as the occipital site, controlling for the sensation of TMS including its lateralization on the head. This location corresponds to the mean location of the representation of the lower limbs in motor cortex^49^. Note that as in the experimental condition, genuine TMS was still applied, but instead to a control site (i.e. not a “sham” condition), controlling for the non-specific artefacts of TMS i.e. the auditory and tactile sensation of receiving stimulation.

### Statistical analysis

All analyses were conducted using the open statistical software R^50^ and the diagrams were created using “ggplot2”^51^. The packages “lme4”^52^ and “lmerTest”^53^ were used to estimate a generalized linear mixed regression model to compare the performance of the two groups in the experimental and the control condition. The mixed model was applied because the individual responses were nested within the subjects and is described in detail in ref.^54^. The model included the digit span performance as dependent variable and two categorical covariables (as well as their interaction term), namely cognitive strategy (verbal = 0; visual = 1) and experimental condition (control = 0; experimental = 1). Because of this dummy coding of the two variables, the “strategy” variable models the mean difference in the control condition between the groups while the “Condition” variable models the difference between control and experimental condition for the verbalizers. The interaction term expresses the two groups’ mean difference in change between control and experimental conditions and constitutes the effect of interest for the research question at hand. The model allowed for random intercepts with effect size coefficients *R*^2^_Marginal_, representing the variance explained by the fixed effects and *R*^2^_Conditional_ representing the variance explained by both fixed and random effects. Effect sizes were estimated using the “MuMIn” package^55^. The individual digits of the series served as a dependent variable (90 responses nested within every subject), so a logistic link function was used to account for the dichotomous responses.

The sample size of *n* = 47 was large enough to detect an effect of *f* = 0.27 with a power of 1 − β = 0.8. This effect size was targeted because it has been related to impairing the preferred processing strategy on DSB performance^17^. All data and the analysis code are provided via the Open Science Framework on https://osf.io/fuw6e/ and can be downloaded.

## Results

**Figure 1:**
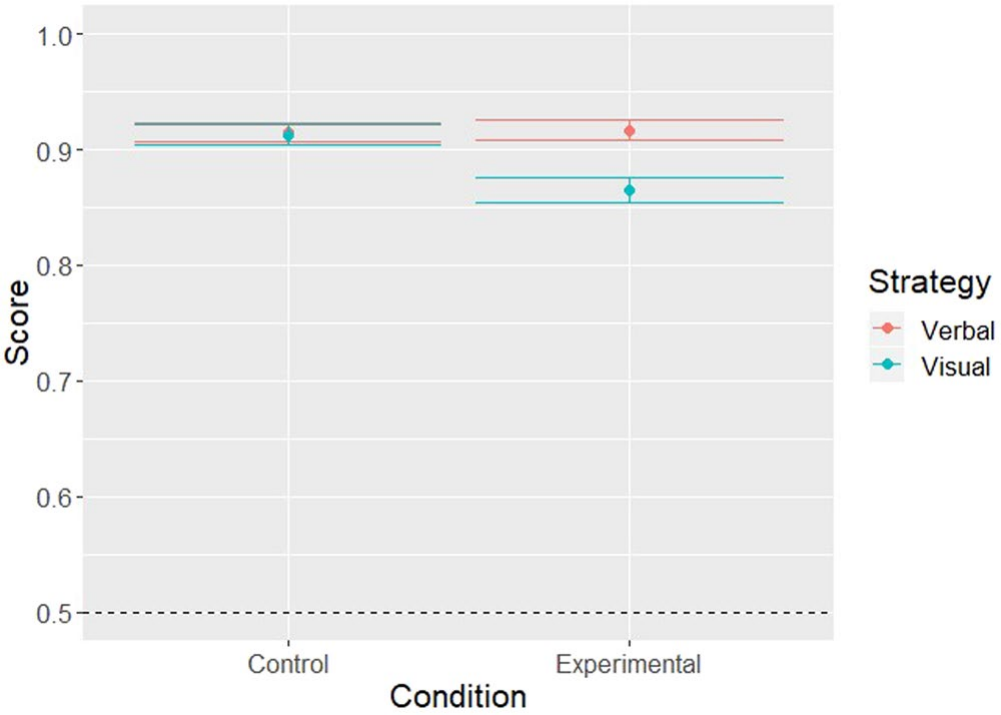
Means and error bars of the percentage of remembered digits for verbalizers and visualizers in the control and the experimental conditions. A Score of 1.00 represents the theoretical maximum of 100% remembered digits. The error bars represent ±1 standard error of the mean. Note that the y-axis starts at 0.5 (dashed line) for an easier comparison of the conditions.

Descriptive statistics for performance in the DSB are depicted in Table 1 for verbalizers and visualizers individually. As depicted in Table 2, the regression coefficient of the covariable “Strategy” did not differ significantly from zero, indicating that the mean performance of verbalizers and visualizers did not differ significantly in the pretest. Moreover, as indicated by the non-significant regression coefficient “Condition”, the verbalizers’ mean performance did not differ significantly between the control and the experimental condition. However, the significant negative regression coefficient of the interaction term indicates a significantly stronger decrease between control and experimental conditions for visualizers compared to verbalizers, which is illustrated in Fig. 1. Fixed and random effects explained *R*^2^_*conditional*_ = 0.25 of the variance in the performance and solely the fixed effects explained *R*^2^_*marginal*_ = 0.02. The mean number of digits remembered in both groups and both conditions are depicted in Fig. 1.

**Table 1:** Descriptive statistics DSB performance.

	Mean	SD	Median	Min	Max
Verbalizers Control	91%	10%	93%	64%	100%
Visualizers Control	91%	10%	94%	58%	100%
Verbalizers Experiment	92%	9%	96%	64%	100%
Visualizers Experiment	86%	10%	89%	58%	100%

SD = Standard Deviation; Min = Minimum Score; Max = Maximum score; Control = Control condition; Experiment = Experimental condition; Values are displayed in percent of correctly remembered digits.

**Table 2:** Mixed regression analysis.

Single Digits	β	SE_β_		*z*	*p*
Intercept	2.81	0.25	—	11.16	<0.001
Condition	0.03	0.16	—	0.17	0.87
Strategy	−0.13	0.34	—	−0.38	0.70
Condition:Experimental	−0.56	0.22	—	−2.61	<0.01

β = Estimated parameter value; SE_β_ = Standard error of the parameter estimate; *z* = *z*-value *p* = Probability of committing a Type-I-Error.

## Discussion

The current investigation was conducted to examine whether an offline virtual lesion applied to the occipital cortex via rTMS would lead to differential effects for verbalizers versus visualizers in the DSB task. It was found that participants using a visual strategy performed significantly worse after occipital stimulation compared to the control condition, while verbalizers showed no significant decrease. It thus indicated that (i) inter-individual differences exist in the processing of optically presented digits, that (ii) participants are aware of their strategies leading to these differences, and that (iii) a visual processing strategy is more dependent on occipital cortex functioning than a verbal processing strategy.

### Cognitive strategies

The involvement of individual processing strategies in the DSB has been indicated long ago: Dunn *et al*. noted that some participants reported to visualize the orally presented digits in order to simply read the internal image during the reproduction phase^13^. They viewed this internal visualization as a “fruitful strategy” for tasks such as the DSB. Further investigating this phenomenon, Hilbert *et al*.^17^ applied a dual task scenario with acoustical and optical stimuli. They showed that additional processing of acoustical stimuli led to inferior performance with optical stimuli when participants adapted a verbal strategy compared to a visual strategy. The finding implies that the processing modality can be chosen rather deliberately and does not directly depend on the input modality^20,21^ but can be translated from verbal to visual and vice versa. This notion is also supported by the present results, since the stimuli in this study were presented optically and the offline lesion was applied to visual areas: the group of participants reporting a verbal strategy was, if at all, only mildly affected compared to visualizers. Moreover, Hilbert *et al*.^14^ found indications for a task-specific choice of processing, implying that participants are in general very aware of their processing strategy. These findings are strongly supported by the present results, as the virtual lesion to the occipital cortex, which is strongly connected to visual working memory processing^21,24,25,35^, strongly deteriorated performance in visualizers but not in verbalizers. Since the participants were categorized as one of the two according to their own self-perception, it is also strongly suggestive that this self-rating is a valid measure of the internal processing strategy.

These results yield important implications for both experimental working memory research and clinical assessment: optical presentation of stimuli does not necessarily tap the visual working memory and acoustical presentation does not exclusively lead to the use of the verbal working memory^17^. Rather, studies of working memory processes should consider the internal processing modality. For example, Hilbert *et al*.^56^ showed that training with working memory tasks including letters lead to improvements in performance in working memory tasks with digits and vice versa (both presented optically). Training with tasks involving patterns of blocks in matrices did not lead to improvements in tasks with letters or numbers and, again, vice versa. A possible explanation is given by the possibility to verbalize and rehearse letters and numbers, which is a strategy often adapted in this kind of task^1^. The arrangement of blocks in matrices, however, is almost impossible to verbalize and rehearse, which would explain the absence of “transfer” between tasks including letters/numbers and blocks.

This is supported by results reported by Hoshi *et al*.^57^ as well as Hilbert *et al*.^14^, indicating that a visual strategy in the DSB is to be related to higher prefrontal cortex activation as well as higher performance. According to Pasternak and Greenlee^35^, sensory cortices are an active component in the system for the retention of information. However, it seems quite plausible that the translation from the visual to the verbal system and vice versa is guided by the prefrontal cortex, which has been shown to play a crucial role in the processing of digit series during verbal working memory activation^58^. This notion would also fit well with identification of this area as the neural correlate of the central executive in Baddeley’s working memory model^2^.

The results also yield implications for clinical practice: a mildly below-average result in the DSB might not be caused by a general working memory deficit but might be due to an impairment of the processes involved specifically in the cognitive strategy of choice. The present results have shown that participants relying on a visual strategy showed an average performance decrease of about five percent when an offline lesion was applied to the occipital cortex, while no such effect was observed for verbalizers with the very same offline lesion. Especially relevant for the clinical application of the DSB, the present findings clearly show that poor performance may be attributable to deficits in the applied cognitive strategy rather than global working memory deficits, which also provides a possible vantage point for the reduction of working memory performance deficits following brain lesions.

### Implications TMS

Unlike purely “correlative” imaging techniques, TMS allows the causal inference that the stimulated brain area makes a critical contribution to a certain behavior. The current results show that a consideration of interindividual differences in baseline strategy can be necessary for establishing a causal role for an area using behavioral effects after TMS. This may apply more broadly with TMS in other systems, for example when studying visuospatial processing in parietal cortex^59^, or response sequence generation and the preSMA: TMS only affects performance at the start of mini-sequences within longer sequences that participants use to “chunk” information^60^.

Different people may achieve similar levels of performance in different ways, and TMS can then be sensitive to this. Although there may only have been few studies looking specifically at the role of interindividual *strategy* differences with TMS, there is currently a strong drive to examine how TMS (or TCS) may covary with other interindividual differences, such as overall accuracy, alertness or putative functional markers. For example, baseline performance modulates TMS effects^33,61,62^ and TMS responses can be partly predicted by individually variable neurophysiological signatures such as alpha power in the EEG^63^ or structural variability within the corpus callosum^64^. Accounting for interindividual variance may be vital for the future use of non-invasive brain stimulation in basic and clinical neurosciences^44,65^, for example by optimizing stimulation profiles according to the brain activity patterns of the participant being stimulated^66^.

More generally, our finding that occipital TMS only affects performance in those who visualize and not those who verbalize indicates that when attempting to determine the function of a brain area with TMS it is not only important to relate neural processes to particular tasks but to the particular strategies used for performing these tasks.

### Limitations and Future Directions

TMS is an interventionist approach assessing the role of disrupting one node at a time within a broader network, lending the method both advantages and disadvantages. Doubtless a wide-ranging network of different areas beyond the occipital cortex plays important roles in working memory and strategy. TMS to various areas outside the occipital lobe has recently been used to briefly reactivate representations of items in working memory^67^. The occipital cortices likely comprise only one part of a widely distributed network.

Behavioral effects of TMS demonstrate that interference with functioning of the stimulated node is relevant for cognition: the key limitation of this interventionist approach is that the change in behavior could theoretically be being implemented by an unusual route that does not mimic the normal functioning of the system^68^. One of the challenges facing brain stimulation is to discriminate between when the consequences are like as opposed to unlike normal cognition^69^. However, the current results inherently go some way to address this limitation by showing that the normal cognitive strategies used by participants help determine the TMS effects.

One key limitation of the use of rTMS as a “virtual lesion”^70^ to induce plasticity is that there is typically a high degree of inter-individual variability in how the brain responds to the TMS, for example with different patterns of responses to different frequencies^71^. It can be expected that the effect of brain stimulation on any particular individual might vary with a whole host of factors, from genetic make-up and cortical geometry to arousal level, age and, gender: explaining such variability may be vital for future accurate metanalysis and for replicability^65^. The current results additionally suggest another source of variability for rTMS effects: individuals may perform the same task in different ways.

These results and their limitations prompt several other studies. For example, one limitation necessary for this design was to separate participants into just two types, whereas it may improve sensitivity to use additional rating scales, where for example participants could describe their strategy not with a binary decision but rather as a continuous confidence rating. Additional methods can also be employed to explore the neural basis of this effect by recording brain activity with fMRI or EEG before and after stimulation^72^, and can indicate whether effects occur through changing synaptic plasticity^73^, and effective connectivity^74^. Neural oscillations at particular frequencies may contribute to these effects and be sensitive to TMS^75^ or tCS^76^. Future work may also compare whether TMS can elicit different effects if targeting specific occipital regions defined from fMRI localiser-based site selection. Here by contrast, we used a stimulation-based site selection method: this can be comparable or even preferable to fMRI guidance in those specific situations (as here) where a behavioural readout of the stimulation (phosphene report) is available^72^. Additionally, different TMS protocols may be expected to produce facilitation rather than inhibition, for example by using higher frequency TMS^77^ or theta burst protocols^78^.

### Conclusion

The present study shows that self-assessment of one’s cognitive strategy can be related to actual cortical processing patterns, which may be disrupted. The findings extend Hilbert *et al*.’s^17^ results, which indicated that different cognitive strategies lead to performance differences in a dual task requiring verbal as well as visual processing, indicating different cortical processing associated with verbal versus visual strategies in the DSB. Within the framework of Baddeley’s working memory model^1^, both the phonological loop and the visuospatial sketchpad can be used in the DSB. rTMS-elicited offline virtual lesions to the occipital cortex, associated with the visuospatial sketchpad, impaired performance only for the group of participants describing their cognitive strategy as visual. This indicates that participants are aware of their processing strategy which, in turn, may provide useful information about the cortical circuits involved in a cognitive task that can be solved with different strategies. Moreover, the study provides further evidence that the stimulus material is not critical for the tapped working memory system but the internal processing strategy, which should be taken into account in laboratory as well as clinical settings.
